# TCR Analyses of Two Vast and Shared Melanoma Antigen-Specific T Cell Repertoires: Common and Specific Features

**DOI:** 10.3389/fimmu.2018.01962

**Published:** 2018-08-30

**Authors:** Sylvain Simon, Zhong Wu, J. Cruard, Virginie Vignard, Agnes Fortun, Amir Khammari, Brigitte Dreno, Francois Lang, Samuel J. Rulli, Nathalie Labarriere

**Affiliations:** ^1^CRCINA, INSERM, Université d'Angers, Université de Nantes, Nantes, France; ^2^LabEx IGO “Immunotherapy, Graft, Oncology,” Nantes, France; ^3^Qiagen Sciences, Frederick, MD, United States; ^4^Centre Hospitalier Universitaire Nantes, Nantes, France; ^5^Department of Dermato-Cancerology of Nantes Hospital, Nantes, France

**Keywords:** TCR sequencing, melanoma, Melan-A, MELOE-1, immunotherapy

## Abstract

Among Immunotherapeutic approaches for cancer treatment, the adoptive transfer of antigen specific T cells is still a relevant approach, that could have higher efficacy when further combined with immune check-point blockade. A high number of adoptive transfer trials have been performed in metastatic melanoma, due to its high immunogenic potential, either with polyclonal TIL or antigen-specific polyclonal populations. In this setting, the extensive characterization of T cell functions and receptor diversity of infused polyclonal T cells is required, notably for monitoring purposes. We developed a clinical grade procedure for the selection and amplification of polyclonal CD8 T cells, specific for two shared and widely expressed melanoma antigens: Melan-A and MELOE-1. This procedure is currently used in a clinical trial for HLA-A2 metastatic melanoma patients. In this study, we characterized the T-cell diversity (T-cell repertoire) of such T cell populations using a new RNAseq strategy. We first assessed the added-value of TCR receptor sequencing, in terms of sensitivity and specificity, by direct comparison with cytometry analysis of the T cell populations labeled with anti-Vß-specific antibodies. Results from these analyzes also confirmed specific features already reported for Melan-A and MELOE-1 specific T cell repertoires in terms of V-alpha recurrence usage, on a very high number of T cell clonotypes. Furthermore, these analyses also revealed undescribed features, such as the recurrence of a specific motif in the CDR3α region for MELOE-1 specific T cell repertoire. Finally, the analysis of a large number of T cell clonotypes originating from various patients revealed the existence of public CDR3α and ß clonotypes for Melan-A and MELOE-1 specific T cells. In conclusion, this method of high throughput TCR sequencing is a reliable and powerful approach to deeply characterize polyclonal T cell repertoires, and to reveal specific features of a given TCR repertoire, that would be useful for immune follow-up of cancer patients treated by immunotherapeutic approaches.

## Introduction

Among solid tumors, metastatic melanoma is a relevant model for immunotherapeutic approaches because of a high immunogenicity, partly due to a high mutation rate, favoring the development of specific T cell immune responses (1). In previous studies, it has been documented that the specific immune response against melanoma is dominated by two vast T cell repertoires specific for the melanoma antigens Melan-A and MELOE-1, which can be selected and amplified from the peripheral blood of HLA-A2 melanoma patients (2, 3). These two antigens share common features regarding their frequent expression in melanoma tumors, the presence of immunodominant HLA-A2 epitopes and of vast specific TCR repertoires in HLA-A2 melanoma patients. Blood frequencies of Melan-A and MELOE-1 specific T cells are respectively around 10^−4^ and 10^−6^ among CD8 T cells. In addition, these T cell repertoires also contain high avidity T cells making these T cell repertoires relevant for a use in adoptive transfer.

For a long time, Melan-A has been regarded as a self-antigen, potentially eliciting a suboptimal T-cell repertoire due to negative selection. However, recently it was reported that the immunodominant HLA-A2 Melan-A_26−35_ epitope is not presented by human medullary thymic epithelial cells, due to a misinitiation of gene transcription (4), and leading to the evasion of central self-tolerance toward this epitope. This finding, together with the strong bias documented in Vα usage (5, 6) could explain the abundance of this specific-T cell repertoire and the presence of high avidity T cells among this repertoire.

On the other hand, MELOE-1 antigen is expressed from a polycistronic RNA, whose expression is controlled by specific transcription factors and epigenetic mechanisms in the melanocytic lineage (7). The translation of MELOE-1 from one of the short ORFs of this RNA is controlled by an IRES sequence, exclusively activated in melanoma cells, conferring to this antigen a strict tumor expression profile (8, 9). Like Melan-A specific T cell repertoire, MELOE-1 specific T cell repertoire also contains high avidity T cells, and is also strongly bias toward the preferential usage of a specific TRAV chain (2), probably contributing to the relative high frequency of MELOE-1 specific T cells in the peripheral blood of HLA-A2 individuals. All these features confer to these two antigen-specific-T cell repertoires interesting properties for a use in adoptive transfer setting in a large subgroup of melanoma patients (HLA-A2), contrary to neo-antigen-based therapeutic personalized strategies.

Based on this, we developed a clinical grade method to select and expand *ex-vivo* Melan-A and MELOE-1 specific CD8 T cells from the blood of HLA-A2 patients. This method, relying on the sorting of specific T cells through the use of HLA/peptide-coated magnetic beads (3), is currently used in the MELSORT clinical trial to treat metastatic melanoma patients (NCT02424916, https://clinicaltrials.gov). This standardized procedure allows the production of fully specific, polyclonal and tumor reactive specific T cells. Nonetheless the diversity of these polyclonal populations has been addressed so far through the use of anti-Vß specific antibodies, and we could document that these populations were composed with various Vß subfamilies, but the number of T cell clonotypes present among a given Vß subfamily remained unknown. Furthermore, the available panel of 24 Vß-specific antibodies does not always cover the entire T cell repertoire of all antigen-specific T cell populations.

We thus took advantage of a recent high throughput TCR sequencing method developed by Qiagen, to fully characterize Melan-A and MELOE-1 T cell populations, selected and amplified according our standardized producing method. We first documented the sensitivity and reliability of this method, and we report here an extensive characterization of Melan-A and MELOE-1 specific T cell repertoires. This analysis reveals a high diversity of these antigen-specific sorted T cells that exhibit common and specific TCR features.

Thus, this method enables the complete and accurate characterization of T cell repertoires that is a main issue for immune follow-up purposes, in adoptive transfer setting, but also for other immunotherapeutic approaches including immune-checkpoint blockade (10).

## Materials and methods

### Melan-A and MELOE-1 specific T cell populations

Peripheral blood mononuclear cells (PBMC) were isolated from 40 mL of blood of HLA-A2 metastatic melanoma patients (Unit of Dermato-cancerology, Nantes hospital) after written informed consent (approval number: DC-2011-1399). PBMC were seeded in 96 well/plates at 2 × 10^5^ cells/well in RPMI 1640 medium supplemented with 8% human serum (HS), 50 IU/mL of IL-2 (Proleukin, Novartis) and stimulated either with 1 μM of Melan-A_A27L_ peptide (ELAGIGILTV) or 10 μM of natural MELOE-1_36−44_ peptide (TLNDECWPA), purchased from Genecust. After 14 days, each microculture was evaluated for the percentage of specific CD8 T lymphocytes by double staining with the relevant HLA-peptide tetramer (from the SFR Sante recombinant protein facility) and anti-CD8 mAb (Clone RPA-T8, Biolegend) using a FACS Canto HTS. Microcultures that contained at least 1% of specific T cells were selected, pooled and sorted with the relevant multimer-coated beads as previously described (3). After a 14-day amplification period on irradiated feeder cells, in presence of PHA-L (1μg/mL) and IL-2 (150U/mL), purity of expanded sorted T cells was assessed by double staining with the relevant HLA-peptide tetramer and anti-CD8 mAb (Figure S1).

### Vß repertoire of specific T cells

Vß diversity of sorted Melan-A and MELOE-1 specific T cell lines was analyzed by labeling with 24 anti-Vß mAbs included in the IOTest Beta Mark TCR V Kit (Beckman-Coulter, IM3497). These cytometric analyses were performed on a Facs Canto II (BD Biosciences).

### T-cell receptor sequencing

Total RNA was extracted from 5 × 10^∧5^ antigen specific T cells using QIAGEN RNeasy Kit. RNA from normal PBMC (purchased from Precision for Medicine) was used as a reference control. 10 or 25 ng of RNA was used to build libraries with the QIAseq Immune Repertoire -T-cell Receptor Panel (Catalog 333705- IMHS-001Z). With this kit, RNA is reverse transcribed with a pool of gene specific primers against the C (constant) region for the T cell receptor alpha, beta, gamma, and delta genes. The reverse transcribed cDNA is then used in a 5′ ligation reaction which adds an oligo which contains one side of sample index and unique molecular index. Following reaction cleanup, a single primer extension is used to capture the T-cell receptor using a pool of gene-specific primers. Resulting captured sequences are amplified and purified using QIAseq beads. The libraries then are sample indexed on the other side by using a unique sample index primer and a universal primer. The final dual sample indexed PCR fragment is purified and then quantitated for abundance using real-time qPCR.

For sequecning, each library was diluted to 4 nM, pooled and denatured. 12 pM of denatured library pool was run on a MiSeq using V3 chemistry for 502 cycles with a pair-end 251 base read.

### Read trimming/clonotype calling

FASTQ files were analyzed in the QIAGEN GeneGlobe Data Analysis Center (https://www.qiagen.com/us/shop/genes-and-pathways/data-analysis-center-overview-page/) using the Immune Repertoire Application The web-based read processing service generates clonotype calls and quantity estimates from reads generated by the QIAseq Immune Repertoire Library Kit. The clonotype calls are generated using the IMSEQ software (11). The main tasks of IMSEQ are to align the reads to model V-region and J-region sequences, extract the CDR3 region sequence, and cluster together highly similar CDR3 sequences that likely came from the same input sample clones. A detailed description of the IMSEQ algorithm is at the following URL: http://www.imtools.org.

### Read processing steps

#### Trim reads

We first trim from the reads the constant regions generated by the enrichment protocol, and move the UMI sequence to the read identifier line.

Trim 3′ end of reads with less than 18 base quality score.
Trim ligation common oligo AGGACTCCAAT from the 3′ end of R1.
Trim uPCR common oligo CAAAACGCAATACTGTACATT from the 3′ end of R2.

The 12 bp UMI sequence is moved from the start of R2 to the FASTQ read identifier comment region of R1 and R2.

#### Down-sample reads

When read depth rises above about 8 to 10 read pairs per UMI, very few new real UMIs are observed, but false UMIs caused by PCR or sequencing errors are observed at an increasing rate. The same is true for CDR3 sequences. To control this over-sequencing error in the UMI and CDR3 sequences, we randomly discard the reads until the remaining reads contain about 8 reads per UMI.

### Merge overlapping R1 and R2 reads

To accommodate IMSEQ requirement for R1 being entirely VDJ sequence, and R2 being V-only, we merge overlapping R1 and R2 and rename them as R1. The reads are then split by gene (TRAC, TRBC, TRDC, TRGC). To accommodate IMSEQ input requirements, we split reads by gene using “cutadapt” search for the C-region sequence between the 5′-most SPE primer and the start of the J-region.

### Trim V region

To accommodate the IMSEQ requirement that reads do not overhang the V-region model sequences, we align the reads to the V-region models using BWA mem, and trim overhang regions (e.g., 5′ UTR regions).

### Run IMSEQ

We run IMSEQ with the following parameters: -ev 0.15 -mq 25 -mcq 25 -ma -qc -sc -scme 2 -sfb.

The model V and J sequences used with IMSEQ can be found here: https://storage.googleapis.com/qiaseq-rna-mmrep/QIAseqRNA_immrep_TCR_model_seqs.zip.

We are using two important features of IMSEQ that are designed to minimize false clonotype calls caused by sequencing error. We are using both the “quality-score clustering” and the “simple edit-distance clustering” with edit distance < = 2 (the IMSEQ default). The main idea here is that reads that contain highly similar CDR3 sequences are putatively from the same clone in the sample, so they are grouped together to generate one CDR3 call, as described previously (11).

### Assign each read to a called clonotype

Although IMSEQ clusters highly similar CDR3 sequences, it does not output detail regarding which reads were clustered together. In part, this is because IMSEQ sometimes counts partial reads, i.e., when a sequence is equal distance from two different CDR3 centroid sequences. To enable UMI counting, we restore the connection between each read and one CDR3 call from IMSEQ, using CD-HIT clustering. We run CD-HIT with the following parameters:

cd-hit-v4.6.8-2017-0621/cd-hit-est-2d -n 5 -g 1 -r 0 -d 0 -G 1 -t 0 -c 0.90 -p 1 -S 2 -S2 2.

### Filter low-evidence clonotypes

To leverage the power of UMI tagging to reduce NGS errors leading to false clonotype calls, we discard IMSEQ CDR3 calls that do not have at least one UMI supported by three reads. Users can set more stringent filters on reported clonotype calls (such as frequency or minimum number of supporting UMIs) depending on application needs.

### Data and statistical analyses

For statistical analyses, clonotypes are defined on the basis of unique amino-acid sequences of CDR3 alpha and CDR3 beta regions. In our set of data, the total number of unique TCR sequences was identical to the number of clonotypes. The standardized residuals of chi-squared is used to determine if a V or J chain is preferentially used in antigen-specific repertoire, score = (observed—expected)^2^/expected, the expected values are calculated from the control sample distribution, the observed values are actually the number of clonotypes using each gene in one given antigen specific repertoire. We considered as significantly used the V or J segment having a score >4.

CDR3 amino acid sequences length has been compared between antigen-specific populations and the control sample using the two-tailed Student's *T*-test. All the calculations were done using R statistical software.

## Results

### TCR diversity of melan-A and MELOE-1 specific T cell populations

We analyzed the TCR diversity of 6 Melan-A and 4 MELOE-1 specific CD8+ T cell polyclonal populations, derived from the specific sorting of HLA-A2 patient PBMC stimulated with the cognate peptides (3). These polyclonal populations were fully specific, as assessed by specific tetramer labeling (Figure S1) and reactive against their target peptides and HLA-A2 melanoma cell lines.

Table 1 summarizes the specific richness of these 10 CD8 T cell populations (numbers of CDR3α and CDR3ß amino-acid sequences, thereafter called “clonotypes,” detected from libraries prepared from 10 to 25ng of total RNA). The RNAseq library includes unique molecular indexes (UMIs) which are added during library construction to remove amplification duplicates and sequencing errors. For our analysis, we considered data where we had at least 1 UMI and 3 reads per UMI to be a true clonotype. For Melan-A-specific T cell repertoires, we observed high numbers of CDR3α and CDR3ß clonotypes with the highest amount of starting RNA, consistent with increased sensitivity in detecting rare clonotypes when starting with more sample. Concerning MELOE-1 specific T cell repertoires, the differences between the number of clonotypes detected with 10 or 25 ng of RNA are either null or rather modest, in accordance with the fact that MELOE-1 specific T cell repertoires are less diverse than Melan-A-specific ones. Thus, the majority of MELOE-1 specific-T cell clonotypes are already detected with 10 ng of starting RNA.

**Table 1:** Number of CDR3 alpha and CDR3 beta clonotypes identified in Melan-A and MELOE-1 specific T cell populations, starting from 10 to 25 ng of total RNA.

	**CDR3 alpha**		**CDR3 beta**	
**Starting RNA**	**10 ng**	**25 ng**	**10 ng**	**25 ng**
**MELAN-A SPECIFIC T CELL POPULATIONS**				
P1	78	140	72	134
P2	61	112	44	100
P3	24	34	14	28
P4	38	46	27	36
P6	22	41	13	26
P7	27	38	13	31
All samples	249	411	183	355
**MELOE-1 SPECIFIC T CELL POPULATIONS**				
P5	49	36	29	29
P8	28	39	23	63
P9	18	20	19	20
P10	62	59	45	41
All samples	157	154	116	153

**Figure 1:**
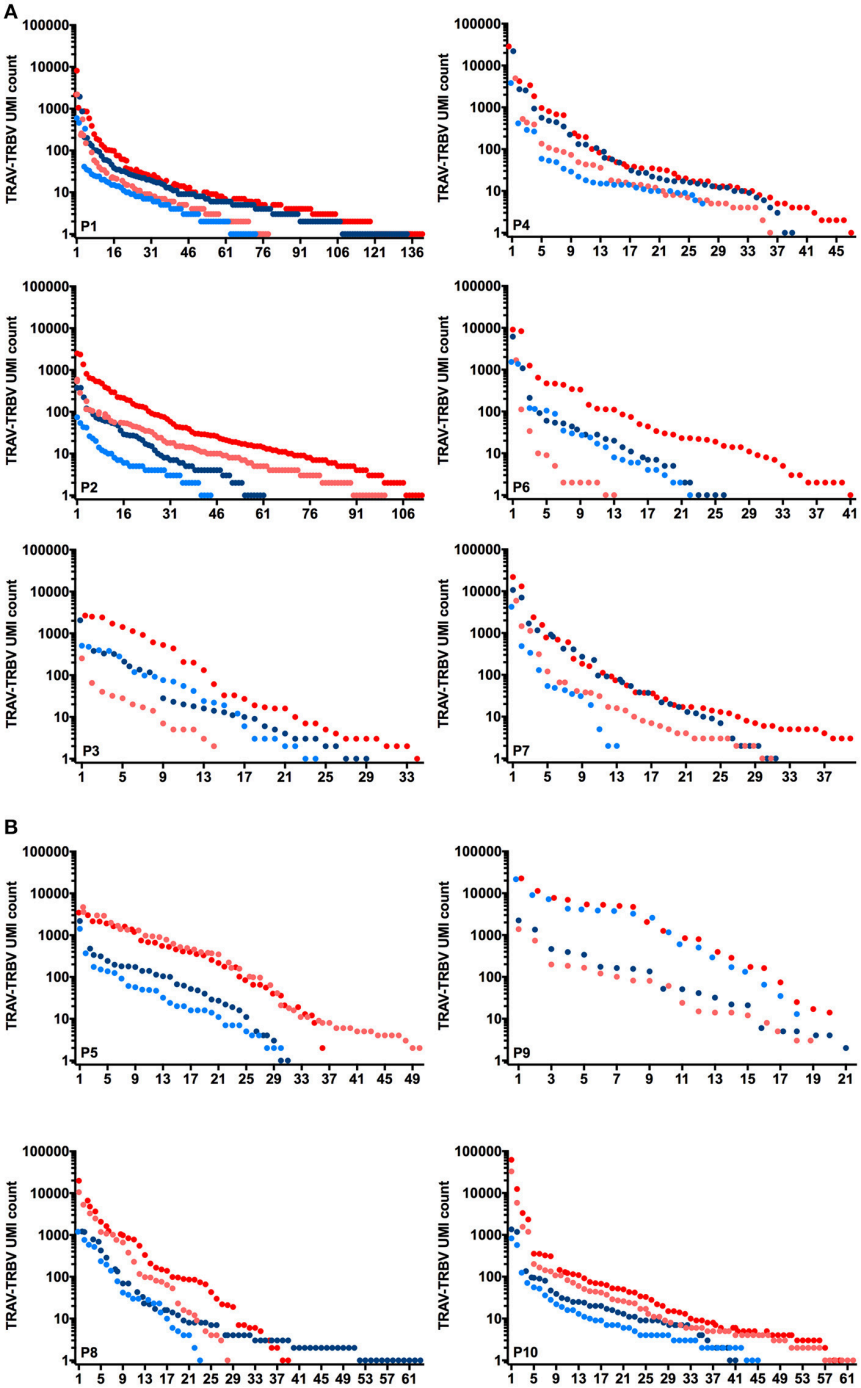
Rank and relative abundance of UMI for individual CDR3α (blue circles) and CDR3ß clonotypes (red circles) identified for 6 Melan-A specific T-cell populations **(A)** and 4 MELOE-1 specific T-cell populations **(B)**, starting from 10 ng (light circles) or 25 ng (dark circles) of total RNA.

Figure 1 illustrates the rank of individual CDR3α and ß clonotypes identified for the 6 Melan-A (1A) and MELOE-1 (1B) specific populations, and the relative abundance of each sequence (number of reads of each sequence associated to a unique UMI), for the two starting RNA quantities. Globally, for Melan-A, and MELOE-1 specific T-cell repertoires, the number of counts for CDR3α (blue circles) and CDR3ß sequences (red circles) was higher when starting with 25 ng (dark circles) vs. 10 ng (light circles) of total RNA. This analysis illustrates the presence of dominant clonotypes within each individual T cell populations, the number of counts for a unique TCR sequence being comprised between 1 and 10^4^. Furthermore, considering the total number of identified clonotypes, we also observed that scarce CDR3 sequences are not identified for some T cell populations with the lowest RNA quantity (Figure 1).

Thus for more diverse populations, higher amounts of RNA will favor the characterization of the complete TCR repertoire, and for less diverse T cell repertoire, RNA quantity will only affect the number of counts for all CDR3 sequences.

### Comparison of TRBV chain frequencies using TCR sequencing or specific antibodies

**Figure 2:**
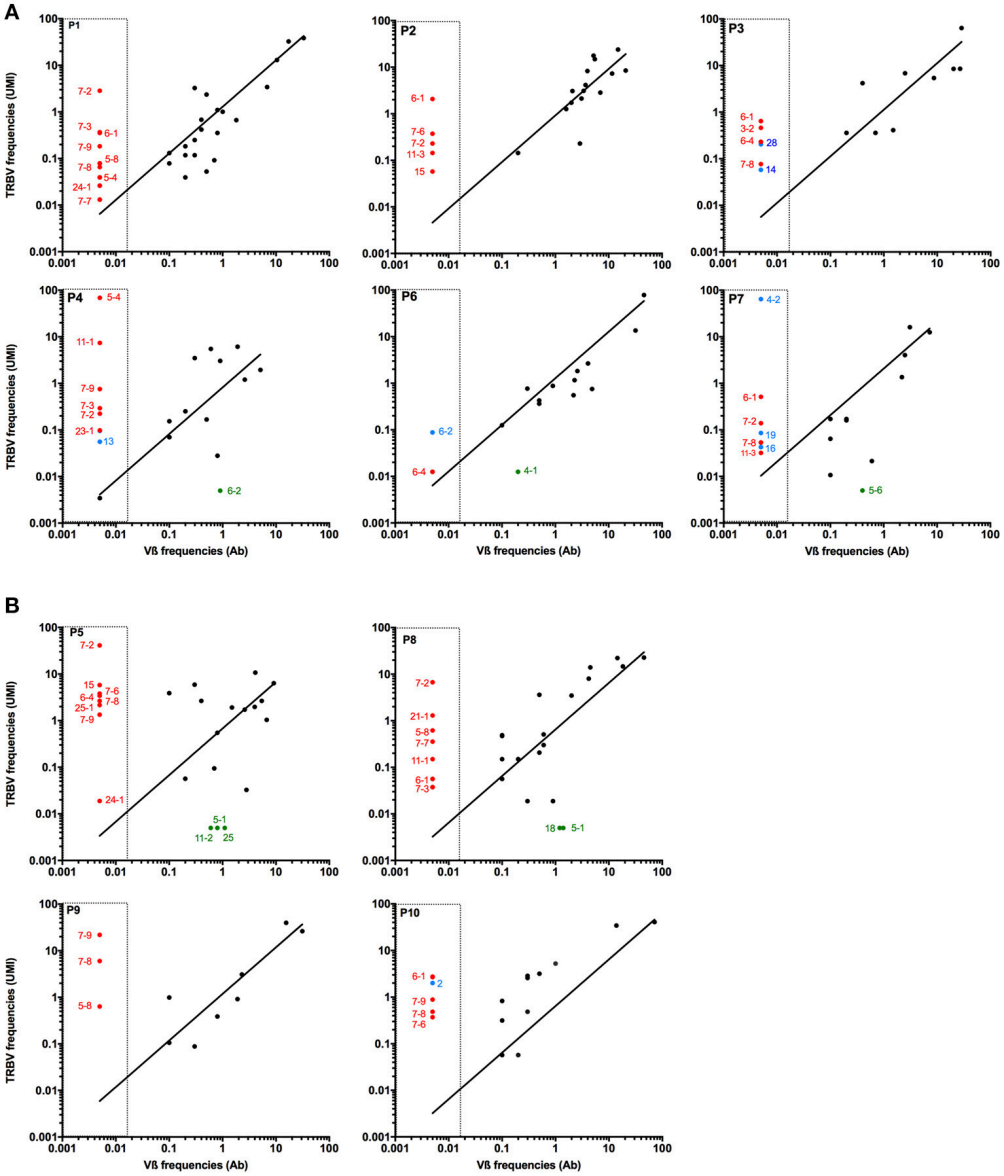
Comparison of Vß chain detection with antibody labeling or high throuput sequencing. The frequencies of Vß subfamilies in each polyclonal Melan-A-specific **(A)** and MELOE-1-specific **(B)** T cell populations were documented through labeling with a 24 Vß-specific antibody panel (X axis). CDR3ß clonotypes identified by sequencing were gathered according to their corresponding Vß chain, and their cumulative frequencies are indicated on the Y axis. Red circles represent Vß chains not covered by the antibody panel. Blue and green circles illustrate respectively Vß chain only detected by TCR sequencing despite the use of a specific Ab, and Vß chain only detected by flow cytometry.

The proportion of T cells expressing a given Vß chain was determined by flow cytometry within the 10 antigen-specific subpopulations, using a panel of 24 Vß-specific antibodies, covering the most frequently expressed Vß chains. Some of the antibodies cross-react with various TRBV subtypes, such as TRBV4-1, 4-2, and 4-3; TRBV6-5, 6-6 and 6-9; TRBV12-3 and 12-4. In order to compare the frequencies of the different TRBV subfamilies detected either by flow cytometry or sequencing approaches, we gather all TRBV sequences potentially detected by a single anti-Vß antibody and calculated their cumulated frequencies. Figure 2 illustrates the correlation between frequencies of TRBV chains detected through the sequencing approach (starting from 25ng of total RNA) and antibody-labeling. TRBV chains for whom there is no available Vß-specific antibody are indicated with red circles. These TRBV chains (especially TRBV6 and TRBV7 subfamilies) are rather frequent within the two antigen-specific T cell repertoires and could only be detected through TCR sequencing. With the exception of these particular TRBV chain the correlation between TRBV frequencies detected with the specific antibodies and cumulated frequencies calculated from sequence counts is satisfying, unless the presence of some outliers only detected through sequencing analysis (blue circles). Generally, the frequency of the concerned TRBV chain is rather low, probably under the detection threshold of specific antibodies. Only the TRBV4-2 chain is detected through TCR sequencing for P7 patient with a high frequency (64%) but is not detected by the specific antibody (Figure 2A). Of note, TRBV4-2 chain is supposed to be detected by an antibody also cross-reacting with TRBV4-1 and 4-3, and we can hypothesize that the reactivity of this antibody against the TRBV4-2 chain is suboptimal. Conversely, some TRBV chains identified through antibody labeling are not detected by the sequencing analyses (green circles on Figure 2). Again, these Vß subfamilies represented only small frequencies, and these discrepancies can be attributed to some degree of cross-reactivity of the concerned antibodies.

This comparison validates the reliability of this TCR sequencing method to estimate the proportion of a specific TRBV chain within a given T cell repertoire. This method is undeniably much more powerful than antibody labeling that leads to underestimate the diversity of a polyclonal population, due to the number of distinct clonotypes within the same TRBV subfamily, to the absence of some TRBV-specific antibodies, and to the cross-reactivity of some specific Vß antibodies.

### TRAV and TRBV usage of melan-A and MELOE-1 specific T cell repertoires

**Figure 3:**
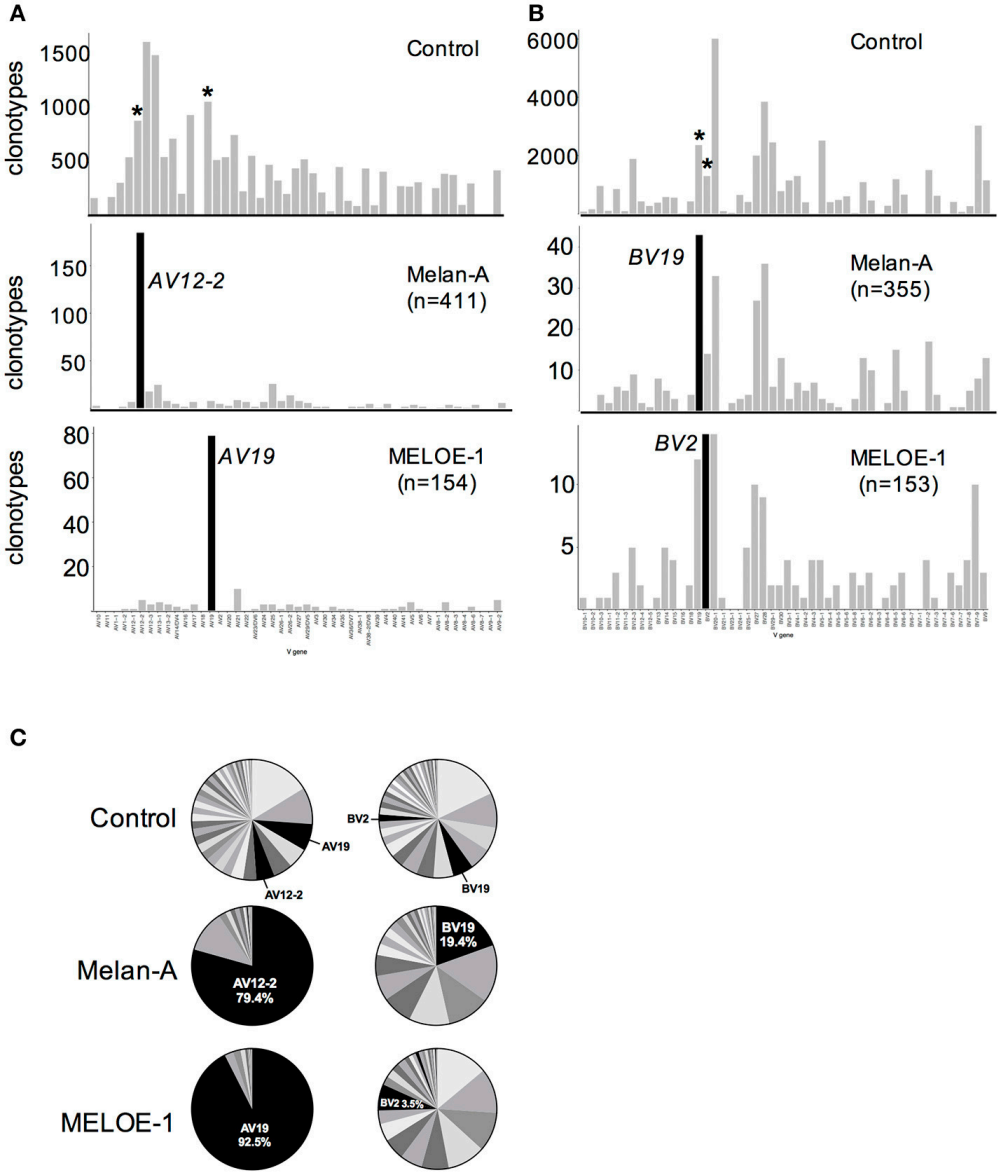
TRAV and TRBV usage of specific-T cell populations. Number of clonotypes using individual TRAV **(A)** and TRBV **(B)** chains in control (upper panels); Melan-A-specific T cell repertoire (sum of the clonotypes originating from the 6 populations; middle panels); and MELOE-1-specific T cell repertoire (sum of the clonotypes originating from the 4 populations; lower panels). Black histograms illustrate TRV chains preferentially used in each T cell repertoire, and the same chains are marked with an asterisk in the control sample. **(C)** Cumulative frequencies (UMI) of each TRAV (left panel) and TRBV (right panel) chains in the control sample, Melan-A specific and MELOE-1 specific T cell repertoires.

Melan-A specific T cell repertoire has been largely studied and it is well known that this T cell repertoire present a strong bias in TRAV12-2 gene usage (5, 6). Likewise, a clear TRAV bias toward TRAV19 chain has also been reported for 18 MELOE-1 specific CTL clones (2). With the aim to increase the statistical value of antigen-specific repertoire analyses and to smooth individual variations, we analyzed TRAV and TRBV usage of all the Melan-A and MELOE-1 clonotypes (originating respectively from 6 and 4 HLA-2 metastatic melanoma patients). The same analyses have been conducted for each individual populations and are illustrated by Figure S2. We clearly confirm the strong recurrent usage of the TRAV12-2 and TRAV19 chains, used respectively by 185/411 Melan-A-specific CDR3α clonotypes (Chi^2^ score value = 34.7) and 79/154 MELOE-1-specific CDR3α clonotypes (Chi^2^ score value = 21) (Figure 3A, left panel). These two chains are also frequently used in the control sample, but their preferential usage by Melan-A and MELOE-1-specific T cells remains strongly significant. This strong recurrence is also remarkable for individual populations (Figures S2A,B, left panels). We also analyzed TRBV usage for these two specific T cell repertoires. A diverse TRBV usage was previously reported for Melan-A specific T cell repertoire (5, 6, 12, 13), nonetheless with some studies highlighting a frequent usage of TRBV20-1, TRBV27, TRBV28, and TRBV19 (14, 15). Here we documented the significant preferential usage of TRBV19 chain for Melan-A specific T cell repertoire, with 43/355 CDR3ß clonotypes (Chi^2^ score value = 6.18). The other described recurrent TRBV chains (TRBV20-1, 27, and 27) were also frequently used by Melan-A specific CDR3ß clonotypes, but this usage was not statistically different from that of the control sample (Figure 3A, right upper panel). At individual population level, although frequently used in each population, TRBV19 usage is only dominant in 2/6 Melan-A specific T cell populations (Figure S2A, right panel).

No preferential TRBV usage has been reported so far for MELOE-1-specific TCR repertoire, and here we documented a significant bias toward the use of the TRBV2 chain, for this TCR repertoire, with 14/153 CDR3ß clonotypes (Chi^2^ score value = 4.58). At individual population level (Figure S2B, right panel), TRBV2 usage is frequent in each T cell population.

We next analyzed the cumulated frequencies of these preferentially used TRAV and TRBV chains within each specific TCR repertoire (Figure 3C). This parameter illustrates the relative abundance of CDR3α and ß clonotypes using these particular TRV genes, within a given repertoire. Within Melan-A and MELOE-1 TCR repertoire, CDR3α clonotypes using respectively the TRAV12-2 and the TRAV19 genes represented almost 80 and 90% of amplified clonotypes, strengthening the crucial role of these TRAV chains in the specificity toward the HLA-peptide complexes. The preponderance of TRAV12-2 and TRAV19 clonotypes, in terms of abundance is also observed in each individual specific-T cell population (Figures S2A,B, inserts on left panel). TRBV19 Melan-A specific clonotypes represented the most abundant ones, with almost 20% of amplified Melan-A specific CDR3ß clonotypes, suggesting that this TRBV19 segment also participates to TCR specificity. Indeed, in individual Melan-A-specific T cell populations, TRBV19 clonotypes are overrepresented in 3/6 Melan-A-specific T cell populations (Figure S2A, inserts on right panel). Conversely, TRBV2 clonotypes represented only 3.5% of total MELOE-1 specific CDR3ß clonotypes, suggesting that the use of a specific TRBV chain is less crucial for MELOE-1 specific T cell repertoire. Indeed, with the exception of P5 patient, TRBV2 clonotypes are not part of the most abundant ones in individual MELOE-1 specific T cell populations.

### TRAJ and TRBJ usage of melan-A and MELOE-1 specific T cell repertoires

**Figure 4:**
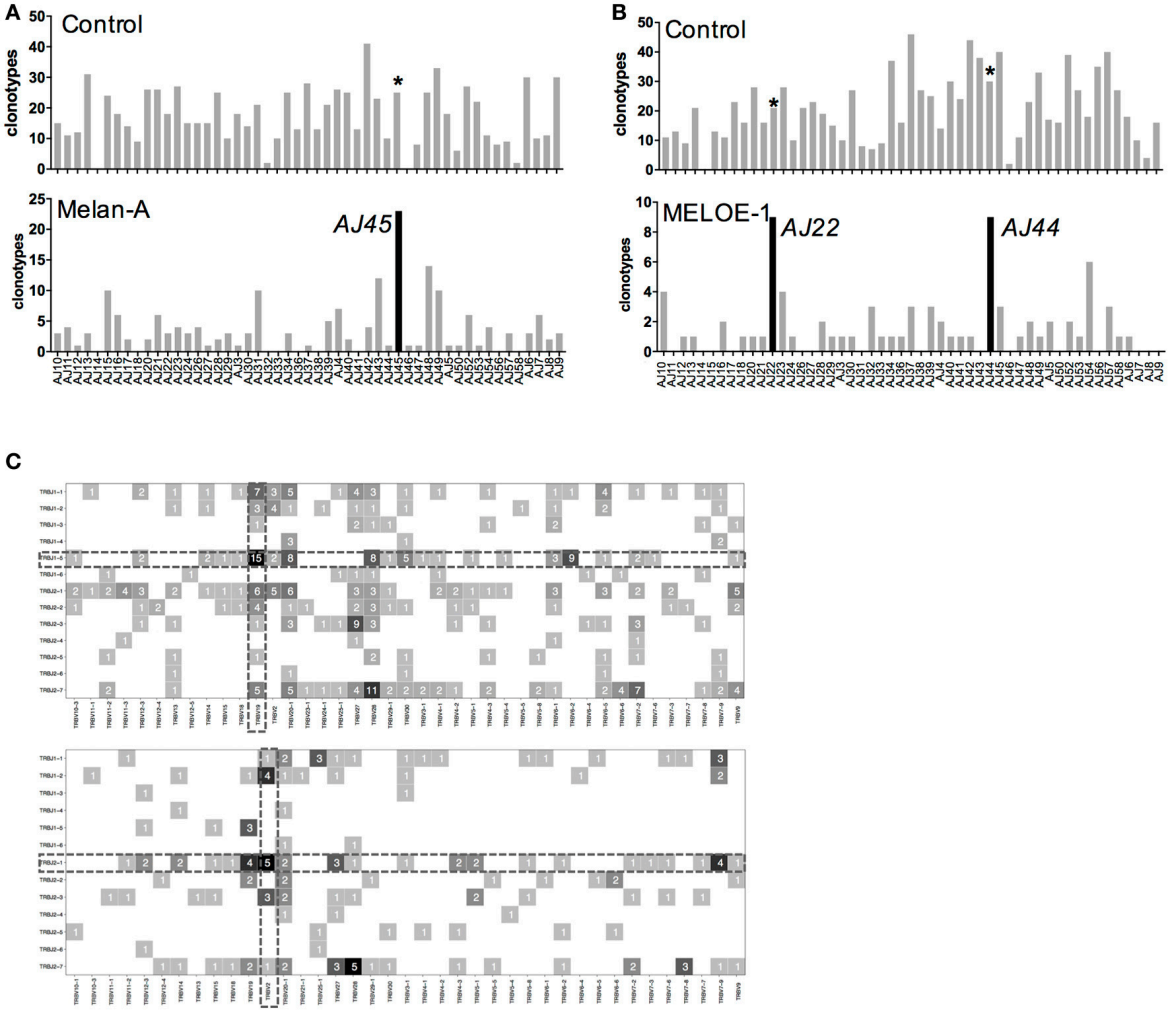
TRAJ and TRBJ usage of specific-T cell populations. **(A)** Number of TRAV-12-2 clonotypes using individual TRAJ in the control sample and Melan-A specific T cell repertoire. **(B)** Number of TRAV-19 clonotypes using individual TRAJ in the control sample and MELOE-1 specific T cell repertoire. Black histograms illustrate TRAJ chains preferentially used in each T cell repertoire, and the same chains are marked with an asterisk in the control sample. **(C)** Heatmaps of TRBV-TRBJ combination usage for Melan-A (upper panel) and MELOE-1 (lower panel)-specific T cell repertoires. Numbers of clonotypes using a given combination are indicated in the corresponding square.

Within the Melan-A and MELOE-1 specific repertoires, we looked for the preferential usage of TRAJ and TRBJ segments (Figure S3) and to particular TRAV-TRAJ and TRBV-TRBJ combinations (Figure 4). We found a significant preferential usage of the TRAJ45 (31/411 clonotypes, Chi^2^ score value = 4.5, Figure S3A) and TRBJ1-5 (67/355 clonotypes, Chi^2^ score value = 8.05, Figure S3B) segments within Melan-A-specific clonotypes. For MELOE-1 specific repertoire, although non-significant, we found some biases in TRAJ usage, with TRAJ22 (9/154 clonotypes, Figure S3A) and TRAJ44 (11/154 clonotypes, Figure S3A) and we also observed a significant preferential usage of the TRBJ2-1 segment within MELOE-1 repertoire (38/153 clonotypes, Chi^2^ score value = 4.3, Figure S3B).

As these Melan-A and MELOE-1 TCR repertoires are strongly biased toward the use of TRAV12-2 and TRAV19 chains, we further investigated whether these dominant TRAV chains were associated with a given TRAJ segment (Figure 4A). For Melan-A-specific repertoire, we confirmed the bias already reported (15, 16) toward the association of the dominant TRAV12-2 chain with the TRAJ45 segment (23/51 TRAV12-2 clonotypes used this segment, i.e., 45%). So far, no specific TRAV-TRAJ association has been reported for MELOE-1-specific T cell repertoire, due to the low number of analyzed T cell clones. Within TRAV19 clonotypes, the preferential use of TRAJ22 (9/51 TRAV19 clonotypes, Chi^2^ score value = 6.02) and TRAJ44 (9/51, Chi^2^ score value = 4.61) segments is significant (Figure 4B).

We also looked for a preferential TRBV-TRBJ association for the two specificities, represented by heatmaps on Figure 4C. For the Melan-A specific repertoire, the significantly preferentially used TRBJ1-5 segment was associated with 21 TRBV chains, that confirms the diversity of Melan-A TRB repertoire. Nonetheless, the most dominant TRBV-TRBJ association was observed with the TRBV19 recurrent BV chain, with 15 CDR3ß clonotypes using the TRBJ1-5 segment among the 43 TRBV19 clonotypes.

For MELOE-1 specific T cell repertoire (Figure 4C, lower panel), the frequently used TRBJ2-1 segment was associated with 21 different TRBV chains, with no obvious specific TRBV-association.

### CDR3 lengths and motif recurrence within melan-A and MELOE-1 specific T cell repertoires

CDR3 sequences were defined according international criteria, beginning by a cysteine residue at the C-terminal end of the V-gene and ending with a phenylalanine residue coded by the N-terminal end of the J segment (17).

**Figure 5:**
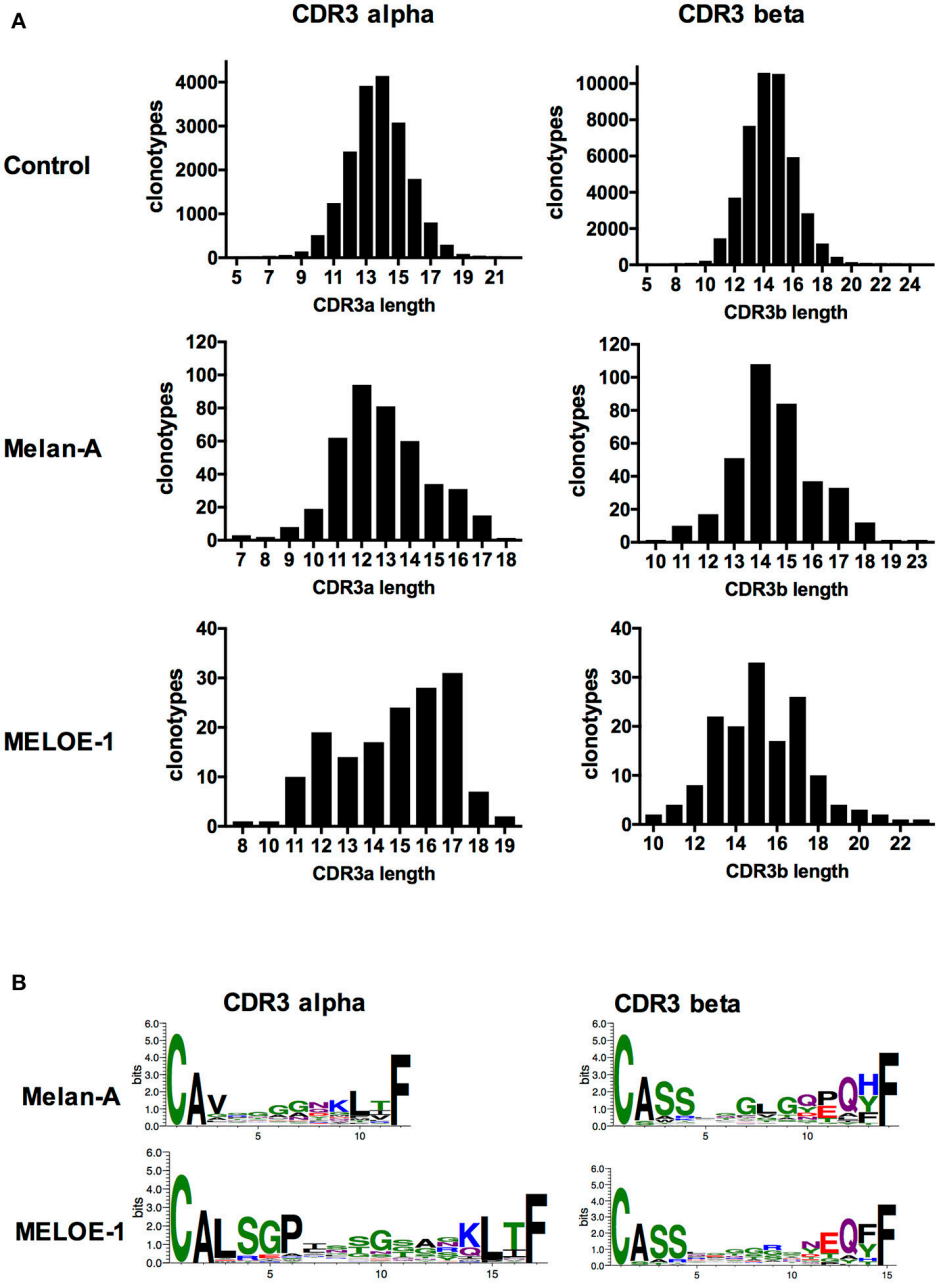
CDR3α and CDR3ß lengths and sequence composition of the most abundant CDR3 lengths. **(A)** Amino acid length distribution of CDR3a (left panel) and CDR3ß (right panel) clonotypes, from the control sample and antigen-specific populations. **(B)** Logotypes of amino acid CDR3α (left panel) and CDR3ß (right panel) composition for the most abundant lengths, for antigen-specific T cell clonotypes.

Lengths of CDR3α and ß sequences of Melan-A and MELOE-1 specific clonotypes were first compared with those of the reference sample (Figure 5A). The average lengths of CDR3α and CDR3ß sequences are between 13 and 14 aa for the control sample. For Melan-A specific repertoire, the mean length of CDR3α is significantly shorter (Student test, *p* = 2.10^−16^), with a length centered on 12 amino acids, and the length of CDR3ß is not different from the control sample. The lengths of CDR3α and ß are more heterogeneous and both significantly longer than those of the reference sample for MELOE-1 specific T cell clonotypes, centered on 17 amino acids for CDR3α and 15 amino acids for CDR3ß (CDR3α: *p* = 2.10^−8^; CDR3ß: *p* = 8.10^−6^).

We further investigated the presence of a conserved motif within these CDR3α and CDR3ß sequences (Figure 5B). For the Melan-A specific CDR3α sequences, we found no clear recurrent motif (upper left). This absence of recurrent motif in the CDR3α sequence is consistent with the fact that the predominant interaction between the TRAV12-2 chain and the HLA-A2/Melan-A peptide is located in the CDR1loop (Gln^31^), and the CDR3α sequence probably does not participate to this interaction (4, 18). Interestingly we also confirmed the presence of the conserved central motif “GLG” for 48/355 CDR3ß sequences (Figure 5B, upper right), that has been already reported (15), suggesting a non-negligible role of CDR3ß in HLA-peptide interaction for this repertoire.

The picture is totally inverted for MELOE-1 specific TCR repertoire, with a strong recurrence of a “GP” motif, formed by non-template added nucleotides, in 58/154 CDR3α sequence (position 5-6 of the CDR3α sequence, Figure 5B, lower left). This motif was previously found in 5/18 MELOE-1 specific CTL clones (2). This suggests a crucial role of the CDR3α sequence for the HLA-peptide interaction. Conversely, no clear recurrent motif was identified in MELOE-1-specific CDR3ß sequences (Figure 5B, lower right).

### Particular features of CDR3 sequences harboring a conserved amino acid motif

**Figure 6:**
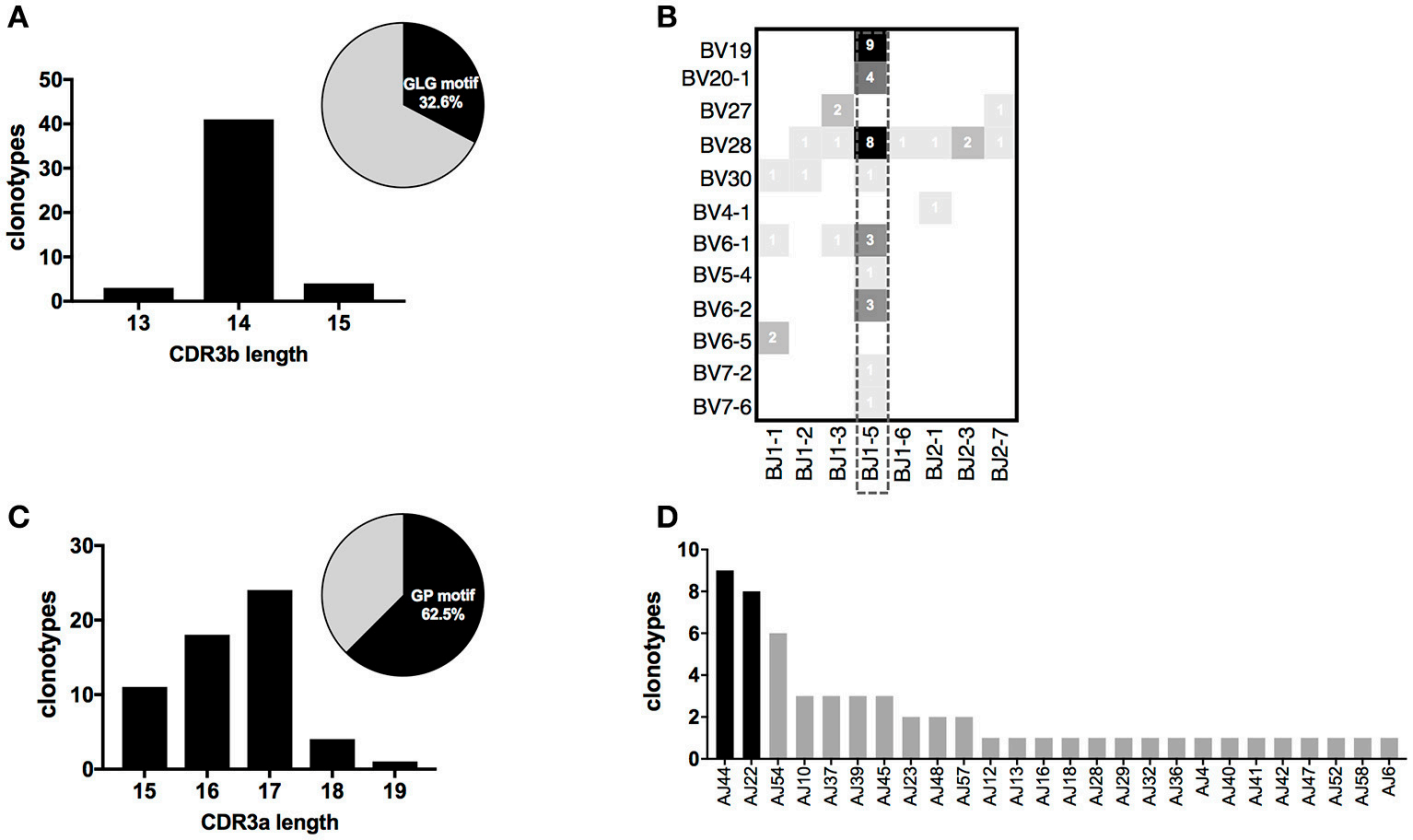
CDR3 length distribution and TRJ usage of clonotypes harboring a conserved motif in their CDR3 sequences. **(A)** CDR3ß length distribution for Melan-A-specific clonotypes with the conserved “GLG” motif. Insert in the figure illustrates the abundance of these 48 clonotypes within the global Melan-A specific repertoire (UMI frequency). **(B)** Heatmap illustrating TRBV-TRBJ association of these 48 Melan-A specific clonotypes. **(C)** CDR3α length distribution for MELOE-1-specific clonotypes with the conserved “GP” motif. Insert in the figure illustrates the abundance of these 58 clonotypes within the global MELOE-1 specific repertoire (UMI frequency). **(D)** TRAJ usage of the 57/58 TRAV19 MELOE-1 specific clonotypes, with the conserved “GP” motif.

The presence of recurrent motifs in CDR3ß and CDR3α clonotypes specific for Melan-A and MELOE-1 antigens prompted us to investigate whether these particular sequences could be associated with specific features. We first analyzed the lengths and the relative abundance of these CDR3 sequences (Figures 6A,C). For Melan-A repertoire, CDR3ß sequences harboring the “GLG” motif were mainly of 14 aa-length (41/48), and these 48 clonotypes represented 32% of total CDR3ß sequences, in terms of abundance (Figure 6A). For MELOE-1 specific repertoire, the lengths of the 58 CDR3α clonotypes harboring the conserved “GP” motif at positions 5–6, are distributed between 15 and 19 amino acids, centered on a length of 17 amino acids. In terms of abundance, these sequences are the majority of MELOE-1-specific CDR3α repertoire, representing more than 62% of total MELOE-1 CDR3α repertoire (Figure 6C).

We further investigated whether these chains harboring a specific motif were associated with particular TRV and TRJ segments. Figure 6B illustrates the use of TRBV and TRBJ segments by the 48 Melan-A-specific CDR3ß clonotypes sharing the “GLG” motif in their sequences. As for the global analysis of TRBV-TRBJ association (Figure 4B), we observed the dominant usage of the TRBJ1-5 segment (31/48 clonotypes), associated with 9 TRBV chains. Of note, the dominant TRBV19 chain is strictly associated with this TRBJ segment for these particular CDR3ß sequences.

All but one (57/58) CDR3α clonotypes harboring the “GP” conserved motif used the TRAV19 dominant chain, that was found preferentially associated with the two previously identified dominant TRAJ segments: TRAJ44 (9 clonotypes) and TRAJ22 (8 clonotypes; Figure 6D).

### Presence of public melan-A and MELOE-1 specific clonotypes

We finally looked for Melan-A and MELOE-1 CDR3α and CDR3ß specific sequences shared between the different populations that originated from distinct metastatic melanoma patients.

**Figure 7:**
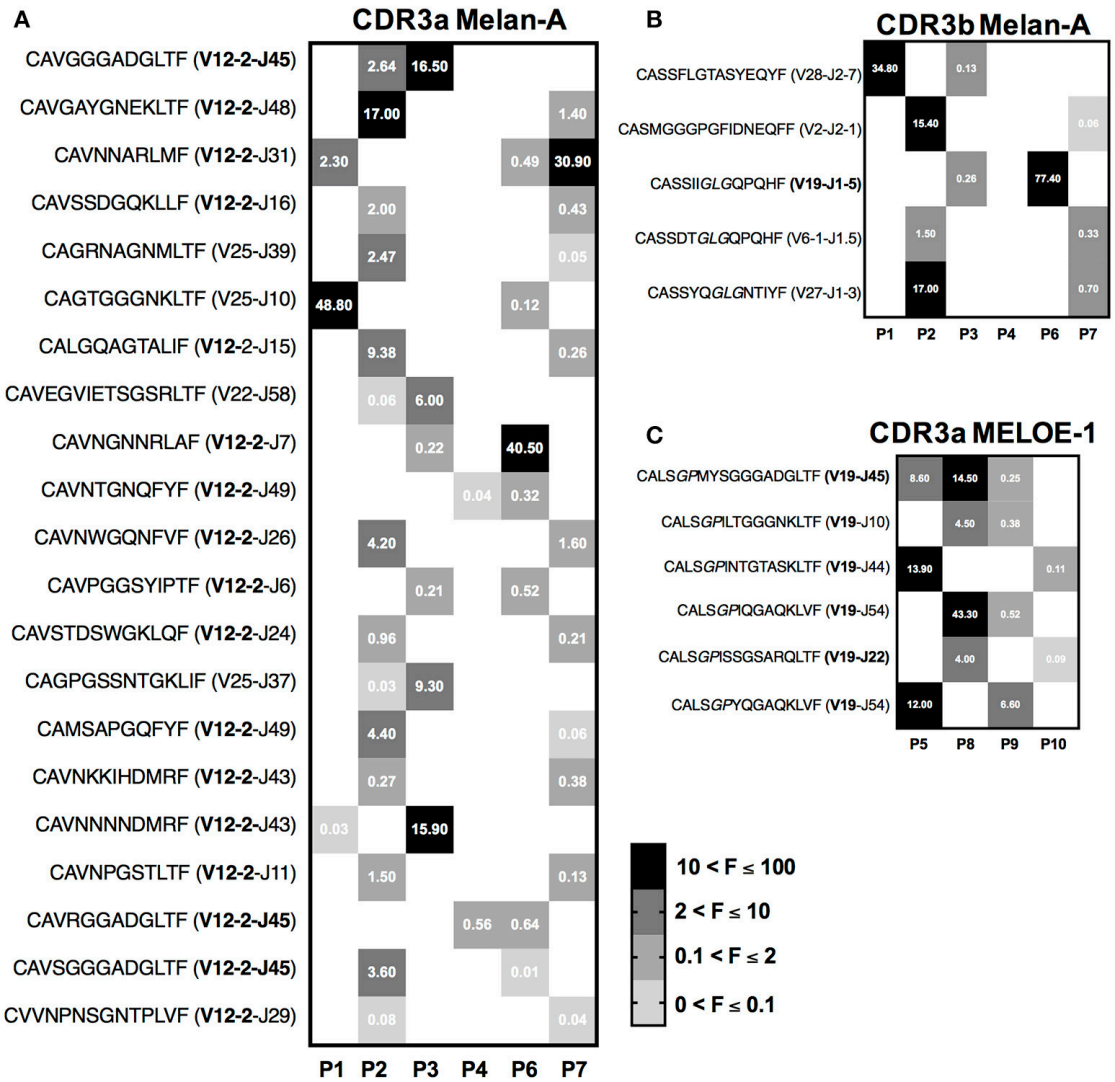
Heatmaps illustrating the sequences and frequencies (UMI) of semi-public CDR3 clonotypes shared by melanoma patients. **(A)** Heatmap of the 21 public CDR3α Melan-A specific clonotypes. **(B)** Heatmap of the 5 public CDR3b Melan-A specific clonotypes. The conserved “GLG” motif is indicated in italics in the sequence. **(C)** Heatmap of the 6 public CDR3α MELOE-1 specific clonotypes. The conserved “GP” motif is indicated in italics in the sequence. The frequencies of each clonotype in individual patients are indicated by the numbers in squares.

Heatmaps on Figure 7 illustrate the sequences and the abundance of each shared CDR3 sequences. Numbers indicated in boxes correspond to the frequency of each clonotype, within a given sample. In order to strengthen the value of our results, we reported here CDR3 clonotypes that have been found as shared between patients with both 10 and 25 ng of starting RNA.

Twenty-one semi-public CDR3α clonotypes were identified for Melan-A specific repertoire (Figure 7A), among them 17 use the TRAV12-2 dominant chain and 3 of them use the preferential association TRAV-12-2/TRAJ45. The majority of these clonotypes are shared between two distinct Melan-A specific T cell populations, and one of them was identified in 3 populations. Frequencies of these shared clonotypes are highly variable in individual samples, but some of them are substantially represented in terms of abundance, reaching 48% of individual CDR3α Melan-A specific repertoire. To a lesser extent, we also identified CDR3ß sequences fully conserved and shared by two distinct Melan-A specific populations (Figure 7B). Among these 5 common CDR3ß sequences, 3 harbored the public “GLG” motif.

Finally, we also performed the same study on the 4 MELOE-1-specific T cell populations, and Figure 7C illustrates the characteristics of the 6 CDR3α sequences that are conserved between 2 and 3 patients. All these sequences use the TRAV19 chain, and thus harbor the public motif “GP” in positions 5–6. As for Melan-A-specific CDR3α shared sequences, the frequencies of these common sequences vary from sample to sample, but can reach up to 43% of individual MELOE-1 repertoire in terms of abundance.

## Discussion

In this study, we analyzed the TCR repertoires of CD8^+^ T cells specific for the immunodominant A2/Melan-A_A27L_ and the A2/MELOE-1_36−44_ melanoma epitopes, originating from the peripheral blood of HLA-A2 melanoma patients, using a recently developed high throughput TCR sequencing method. This method, based on UMI (Unique molecular indexes) technology strongly reduces PCR duplicates and amplification bias, that are major issues in current RNAseq workflows. These molecular barcodes allow the counting of original transcript levels instead of PCR duplicates, thereby enabling digital sequencing and resulting in unbiased and accurate gene expression profiles (19). TCR sequencing was performed on 6 Melan-A and 4 MELOE-1-specific CD8^+^ T cell populations, amplified *in vitro* after sorting with HLA-peptide coated magnetic beads (3, 10). Libraries prepared from 10 to 25 ng of total RNA revealed that the initial quantity of material is an issue to reveal the entire diversity of the most polyclonal populations, especially for clonotypes present at the lowest frequencies. Indeed, the total number of sequenced CDR3α and CDR3ß clonotypes (cumulated from all populations) increased by nearly half for Melan-A specific repertoire, when starting with the highest RNA quantity. For the less diverse MELOE-1 specific repertoire, the number of total CDR3α and CDR3ß clonotypes is rather similar with the two RNA starting quantities. As expected, and illustrated by Figure 1, the highest quantity of starting material allows the detection of the highest number of low frequency clonotypes.

In this study, we also assessed the reliability of this TCR sequencing method comparing the frequencies of CDR3ß clonotypes sharing the same TRBV chain, detected either through TCR sequencing or labeling of polyclonal T cells with Vß-specific antibodies (Figure 2). For most TRBV chains, for which a specific antibody is available, there was globally a good correlation between the cumulated frequencies obtained from TCR sequencing and the fraction of Vß positive cells detected by cytometry. Nonetheless, we observed some outliers, detected at low frequencies either by TCR sequencing, or antibody labeling. This could be explained by the lower sensitivity of antibody labeling and to some degree of cross-reactivity of some specific antibodies (Figure 2). Globally, this TCR sequencing method is a very powerful, sensitive and reliable method to reveal the diversity of polyclonal T cell populations.

The quality of obtained results was also assessed by the confirmation of specific features already described for Melan-A specific T cell repertoire. First, we confirmed a very strong bias in TRAV usage, with the dominant use of TRAV12-2 for Melan-A specific repertoire. This dominance has been widely explored in Melan-A-specific T cells from different origins (TIL, T cell clones originating from tumors or blood from melanoma patients or HLA-A healthy donors), (5, 6, 20). This TRAV12-2 recurrence occurs for T cells specific for the natural epitope Melan-A_26−35_ almost all cross-reactive with the heteroclitic Melan-A_A27L_ peptide, despite the fact that TCR engagement of these two peptides differs in terms of the strength of the interaction (18, 21). Indeed, it has been demonstrated that the TCR is extremely sensitive to minor alterations in peptide conformation and that the use of heteroclictic peptide can skew the natural specific T-cell repertoire (22). Therefore we cannot formally assert that observed features for Melan-A_A27L_-specific T cell repertoire would be observed in the same proportions for Melan-A_26−35_ specific T cell repertoire, although a high degree of similarity between the two repertoires has been reported in structural studies. Indeed, structural analyses of the interaction between HLA-peptide complexes and both Melan-A_26−35_- and Melan-A_A27L_ specific TCR revealed a strong interaction between the TRAV12–2 CDR1 and the Melan-A _26−35_ peptide, presented in the HLA-A2 molecule (4, 23), the CDR1 loop acting as the classical CDR3 loop considering peptide contacts. This unusual TCR binding mode (involving a germline-encoded region) has been proposed to explain the high frequency of naive Melan-A-specific precursors. Supporting this hypothesis, two other T cell repertoires, with a very high frequency of naive precursor, also exhibit a strong bias in TRAV12-2 usage, with a major role of the CDR1 loop: the T cell repertoire specific for the HTLV-1/A2 dominant epitope, (24) and for Yellow fever/A2 dominant one (23). We also confirmed the dominant usage of TRAJ45 segment (16), in the whole Melan-A specific T cell repertoire and even more significant for the TRAV-12-2 expressing clonotypes (Figure 4A), also suggesting a combinatorial constraint favoring the association of these two segments for Melan-A_A27L_ repertoire.

A diverse TRBV usage has been reported for Melan-A specific T cell repertoire (5, 6), nonetheless with the recurrence of some TRBV chains, such as TRBV19, BV20-1, BV27 and BV28 (14, 15). It has been documented that TRBV repertoires -specific for the natural and analog Melan-A peptides were overlapping, nonetheless with the preferential usage of TRBV19 by Melan-A_A27L_ specific TCR (16). Our results confirmed this bias, with 43/355 clonotypes using the TRBV19 chain, representing almost 20% of amplified clonotypes. The TRBV20-1, BV27, and BV28 chains are also frequently used, but as these chains are very frequent in the control population, their preferential usage in Melan-A-specific repertoire does not appear significant. Results also confirmed the preferential usage of TRBJ1-5 segment (Figure S3), that was also found strongly associated with the dominant TRBV19 chain (15/43 clonotypes, Figure 4B). A recurrent usage in TRAJ1-5 segment had been previously reported (15), with a preferential combination with TRBV28 chain. In our study, the combination TRBV28-TRBJ1-5 is also present although less dominant (8/36 TRBV28 clonotypes used this segment) than the TRBV19-TRBJ1-5.

The analysis of CDR3α and ß amino acid composition revealed no specific features concerning CDR3α sequence, but a recurrent central motif “GLG” in CDR3b region (Figure 5B) already documented (15, 16). The resolved TCR/HLA-A2-Melan-A_A27L_ structure revealed that the residues “LG” of the CDR3b made interactions with the Ile^P7^ of the Melan-A_A27L_ peptide. Thus, the CDR3ß loop may contribute to the stability of the TCR-Melan-A_A27L_ complex (18). Interestingly, TCR clonotypes harboring this specific motif represented more than 30% of total CDR3ß clonotypes (Figure 6A), strengthening the role of this conserved motif for TCR/HLA-peptide interactions. CDR3ß regions harboring this specific motif are mainly of 14 aa-length (41/48 clonotypes), with a clear biased usage of TRBJ1-5 segment (Figure 6B). Of note, all the TRBV19 clonotypes harboring this specific motif were associated with this segment, also dominant within TRBV28 clonotypes, in accordance with previous report (15). Our results thus confirmed the existence of a conserved “GLG” amino acid motif in CDR3ß sequences of Melan-A-specific T cells, together with the preferential usage of TRB1-5/TRBV19 combination, and to a lower extent of TRBJ1-5/TRBV28. This strengthens the hypothesis that, besides the well-documented role of the CDR1 region of TRAV12-2 chain, the role of TRB chain, and especially that of the CDR3ß region is far from anecdotal for the sharpness of TCR interaction with Melan-A peptides.

We perform the same analysis on MELOE-1 specific T cell repertoire, that has been far less extensively characterized. Indeed, we reported before that MELOE-1 specific T cell repertoire was also a vast T cell repertoire in HLA-A2 healthy donors and melanoma patients, and that MELOE-1 specific T cells were strongly biased toward TRAV19 usage (2). This initial study was performed on 18 specific T cell clones of diverse origins, and here we clearly confirmed this strong bias on 79/154 clonotypes from 4 different melanoma patients (Figure 3). These TRAV19 clonotypes represented more than 90% of total clonotypes, in terms of frequency, strengthening the crucial role of this TRAV chain in the specificity toward the HLA-2-MELOE-1_36−44_ complexes. This TRAV19 chains appears preferentially associated with TRAJ44 and TRAJ22 segments (Figure 4B). The analysis of CDR3α sequences reveals interesting features. First, the lengths of CDR3a appeared significantly longer than in the control population, with a mean-length situated around 16–17 amino acids. Furthermore, this analysis also revealed the presence of a very highly conserved motif at the beginning of the CDR3α sequence: CALSGP, in which GP residues are encoded by the diversity. The presence of this conserved motif was previously observed in 12/18 of MELOE-1 specific T cell clones (2), and suggested that, contrary to that described for Melan-A repertoire, the CDR3α region of these TCR could be a key player in the specific interaction with MELOE-1_36−44_ peptide. Of note, all but one clonotypes harboring this specific motif, and representing 62% of total clonotypes in terms of frequency, used the TRAV19 chain (Figure 6C). The dominant length of these clonotypes is of 17 amino acids, and in this subgroup, the TRAV19 chain is mainly associated with TRAJ44 and TRAJ22 segments. This study also revealed a preferential usage of the TRBV2 chain with 14/153 clonotypes, nonetheless representing only 3.5% of expanded clonotypes (Figures 3B,C). Thus TRBV chain may be less crucial in conferring TCR specificity, also confirmed by the absence of any clear conserved motif in CDR3ß sequences (Figure 5B). The most significant feature concerning TRB chain for MELOE-1 specific repertoire was the dominant usage of the TRBJ2-1 segment (38/153 clonotypes), associated with 21 different TRBV chains. This suggest, that TRBJ segment, rather than TRBV chain could be involved in TCR-peptide interaction.

Overall, these data suggest two different structural hypotheses that could explain the high frequencies of Melan-A and MELOE-1 specific T lymphocytes, based either on a specific role of the germline encoded CDR1α and the somatically rearranged CDR3ß regions for Melan-A T cell repertoire, or based on probable interactions within the somatically rearranged CDR3α region, for MELOE-1 specific T cell repertoire, as suggested by the presence of the highly conserved “GP” motif in TRAV19 chain, and also possibly involving the TRBJ2-1 segment.

Based on these particular features, we investigated the presence of public or semi-public clonotypes shared by the different patients from whom these T cells have been derived. Such CDR3α clonotypes have been previously described for Melan-A specific repertoire (5, 6, 16), as also reported for T cells submitted to chronic exposure to antigens (25, 26). Here we found 21 CDR3α semi-public sequences, shared at least by 2 patients (one shared by three patients). Among these clonotypes, 17/22 use the TRBV12-2 chain, and some were highly frequent in individual Melan-A TCR repertoires. Interestingly, 2 of these TRAV12-2 public clonotypes (CAVNNARLMF and CAVGGGADGLTF) have been previously identified from the blood of patients either vaccinated with the natural or the analog Melan-A peptides (16). Nonetheless, no conserved motif was identified within these semi-public clonotypes, strengthening again the fact that CDR3α chain is not involved in TCR-peptide interactions (Figure 7). We also observed 5 semi-public CDR3ß sequences, among them 3 harboring the conserved “GLG” motif, and one of these clonotypes (CASSFLGTASYEGYF) being previously reported has a public one (16).

No public CDR3 sequences have been described so far for MELOE-1 specific T cell repertoire, and here we documented the existence of 6 CDR3α sequences shared by 2 distinct melanoma patients. All of them were associated with the TRAV19 chain and harbored the conserved “GP” motif, previously identified. However, no public CDR3ß sequences were found for MELOE-1 repertoire. This final result supports the potential crucial role of CDR3α region in conferring the specificity toward MELOE-1 epitope, and could also explain the lower frequency of MELOE-1 specific T cells (around 10^−5^ in CD8^+^) compared to Melan-A specific ones (around 10^−4^), whose TCR specificity is mainly conferred by the TRVA12-2 germline encoded CDR1 loop.

Globally this study highlighted common and specific features between T cell repertoires specific for two melanoma antigens, that are relevant targets for immunotherapy. We cannot formally rule out the possibility that *ex-vivo* peptide stimulation, sorting and amplification steps could introduce some biases in the relative abundance of some clonotypes harboring particular features. Nonetheless, results obtained about the dominance of TRAV12-2 and TRAV19 usage, and on specific features of Melan-A-specific CDR3beta sequences, confirmed already reported results, some of them obtained without any culture biases. Therefore, it appears quite plausible to suggest that the new described T cell repertoire features could arise at least partly from *in vivo* amplified T cell repertoires. Beyond these specific results, high throughput TCR sequencing approaches provide reliable and exhaustive T cell repertoire analyses, and will be a real asset to monitor immunotherapy-treated patients, with the aim to improve immunotherapeutic treatments.

## Ethics statement

This study was performed in accordance with the declaration of Helsinki and after approval by an institutional review board (IRB: Nantes ethic committee). Peripheral blood mononuclear cells (PBMC) were isolated from HLA-A2 metastatic melanoma after written informed consent (approval number: DC-2011-1399).

## Author contributions

SS, SR, and NL designed the experiments and wrote the manuscript. SS, ZW, AF, and VV performed the experiments. SS, JC, ZW, SR, and NL analyzed the data and prepared the figures. NL, SS, and SR supervised the project. AK and BD provided melanoma patients blood samples and regulatory issues. FL revised the manuscript. All authors read the manuscript carefully.

### Conflict of interest statement

The authors declare that SR and ZW are employed by QIAGEN, however the research was conducted in the absence of any potential conflict of interest. The remaining authors declare that the research was conducted in the absence of any commercial or financial relationships that could be construed as a potential conflict of interest.
