# Supplementary material for: TCR Analyses of Two Vast and Shared Melanoma Antigen-Specific T Cell Repertoires: Common and Specific Features
Source: Front Immunol. 2018 Aug 30;9:1962. doi: 10.3389/fimmu.2018.01962 (PMC6125394; doi:10.3389/fimmu.2018.01962)
Supplement: Supplementary file 1 [file Data_Sheet_1.PDF]

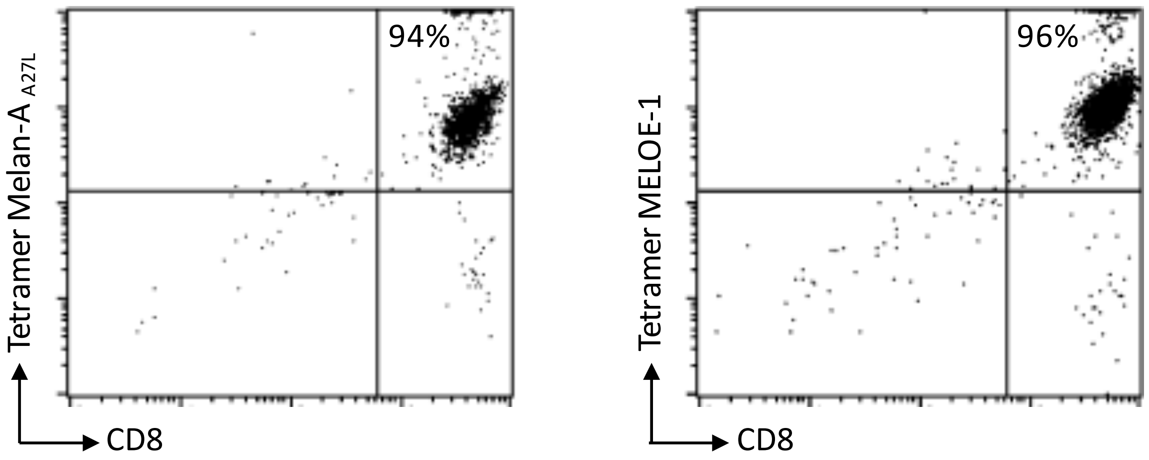

Figure S1 : representative examples of the purity of sorted and amplified Melan-A (left panel) and MELOE-1 (right panel) specific T cell populations. Purity was assessed through double labelling with CD8 antibody and specific tetramer, and analyzed by flow cytometry.
