# Supplementary material for: TCR Analyses of Two Vast and Shared Melanoma Antigen-Specific T Cell Repertoires: Common and Specific Features
Source: Front Immunol. 2018 Aug 30;9:1962. doi: 10.3389/fimmu.2018.01962 (PMC6125394; doi:10.3389/fimmu.2018.01962)
Supplement: Supplementary file 2 [file Data_Sheet_2.pdf]

## Figure S2

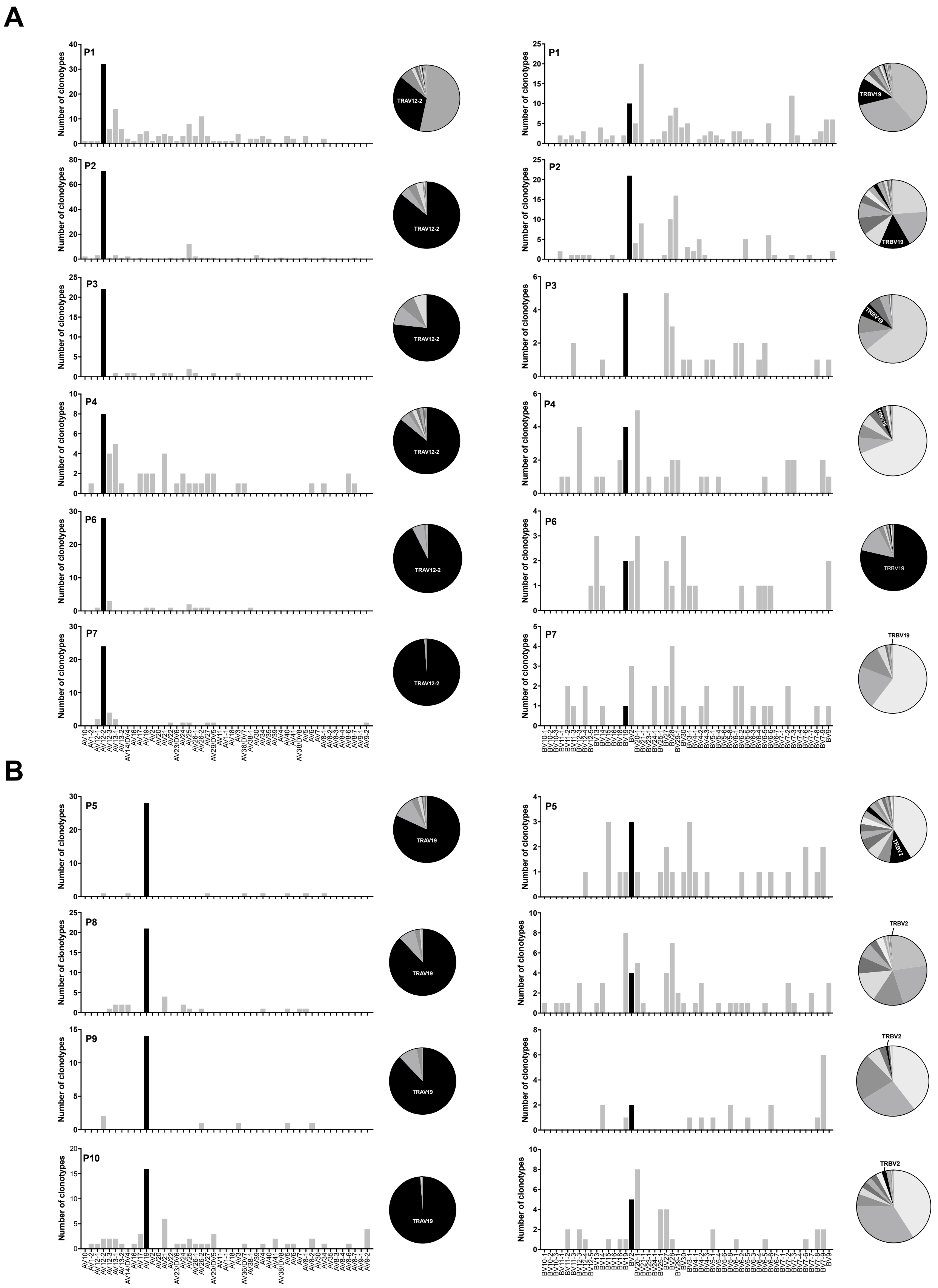


Figure S2: TRAV and TRBV usage of individual specific-T cell populations. Number of clonotypes using individual TRAV (left) and TRBV (right) chains in individual Melan-A-specific T cell populations (A) ; and individual MELOE-1-specific T cell populations (B). Black histograms illustrate TRV chains preferentially used in antigen-specific T cell repertoires. Pies represent cumulative frequencies (UMI) of each TRAV (left panel) and TRBV (right panel) chains in Melan-A specific (A) and MELOE-1 (B) individual specific T cell repertoires.
