# Supplementary material for: TCR Analyses of Two Vast and Shared Melanoma Antigen-Specific T Cell Repertoires: Common and Specific Features
Source: Front Immunol. 2018 Aug 30;9:1962. doi: 10.3389/fimmu.2018.01962 (PMC6125394; doi:10.3389/fimmu.2018.01962)
Supplement: Supplementary file 3 [file Data_Sheet_3.PDF]

Figure S3

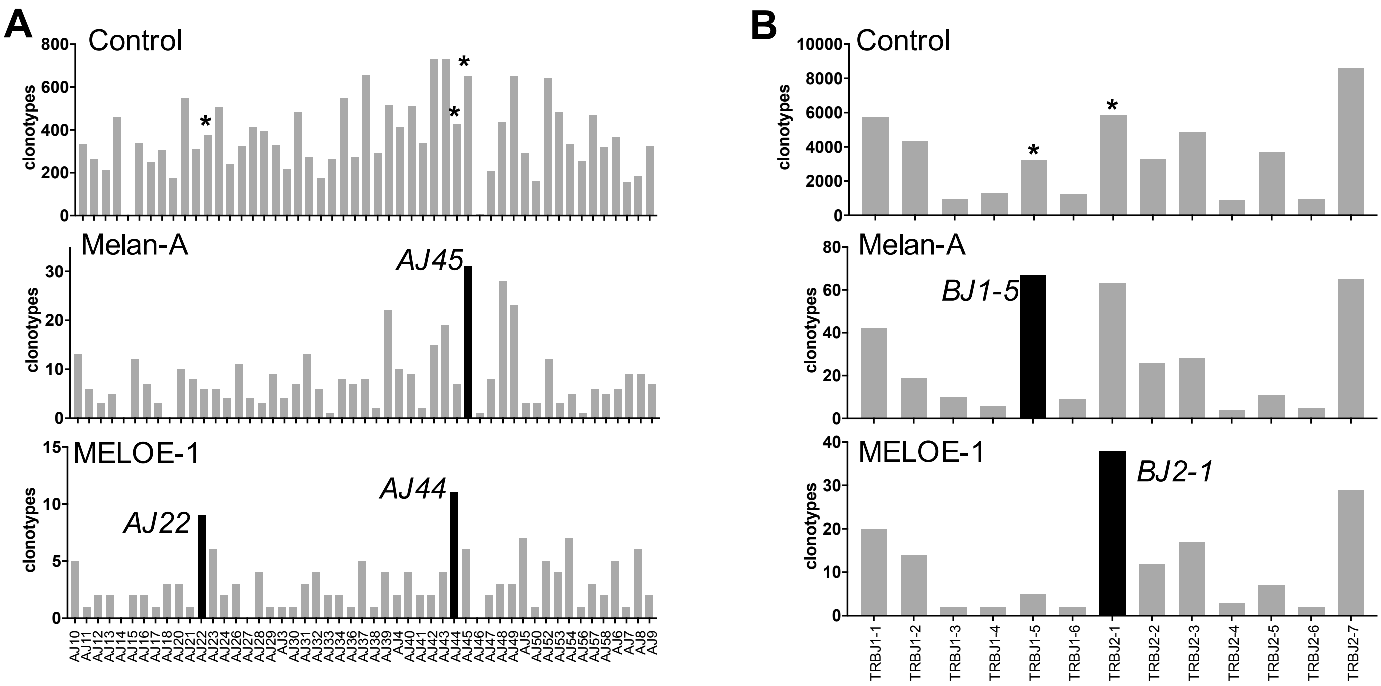

Figure S3: TRAJ and TRBJ usage of specific-T cell populations. Number of clonotypes using individual TRAJ (A) and TRBJ (B) chains in control (upper panels); Melan-A-specific T cell repertoire (sum of the clonotypes originating from the 6 populations) (middle panels); and MELOE-1-specific T cell repertoire (sum of the clonotypes originating from the 4 populations) (lower panels). Black histograms illustrate TRJ chains preferentially used in each T cell repertoire, and the same chains are marked with an asterisk in the control sample.
